# Helicobacter pylori infection among patients presenting with dyspepsia at a primary care setting in Cameroon: seroprevalence, five-year trend and predictors

**DOI:** 10.1186/s12879-019-3677-0

**Published:** 2019-01-08

**Authors:** Jeannine A. Aminde, Guisilla A. Dedino, Calypse A. Ngwasiri, Kingsley S. Ombaku, Cedric A. Mahop Makon, Leopold Ndemnge Aminde

**Affiliations:** 1 Faculty of Health Sciences, University of Buea, Buea, Cameroon; 2 Etoug-Ebe Baptist Hospital, Yaounde, Cameroon; 3 Regina Pacis Hospital, Mutengene, Cameroon; 4 Clinical Research Education, Networking & Consultancy (CRENC), Douala, Cameroon; 5 Bamendjou District Hospital, Bamendjou, Cameroon; 6 Wum District Hospital, Wum, Cameroon; 7 Faculty of Medicine, School of Public Health, The University of Queensland, Brisbane, Australia

**Keywords:** Helicobacter pylori, Seroprevalence, Trends, Primary care, Cameroon

## Abstract

**Background:**

Almost half the world’s population is infected with Helicobacter pylori (*H. pylori*) with the highest reported prevalence from Africa. This infection is associated with several morbid gastrointestinal conditions. Understanding the trends in seroprevalence and the factors associated with *H. pylori* seropositivity in dyspeptic persons can provide a guide for public health policies.

**Methods:**

This was a retrospective study, carried out with outpatient records of Wum District Hospital (WDH) from January 2012 to December 2016. We reviewed records of all patients for whom a *H. pylori* serology test was requested. The Cochran-Armitage trend test and multiple regression models were used to explore seroprevalence trends and predictors of seropositivity respectively.

**Results:**

We included 451 records, 63.6% (*n* = 287) were female. The mean age of the study population was 40.7 years, and the overall *H. pylori* seroprevalence was 51.5% (95% CI: 47–56%). The use of recommended eradication regimen appears to be low and declining. On average, *H. pylori* seroprevalence declined by 6.8% annually (*p* < 0.0001). Occupational status independently predicted seropositivity, with students having lower odds of being seropositive than employed persons (aOR = 0.09, 95% CI: 0.02–0.49, *p* = 0.016).

**Conclusion:**

Despite decreasing trends, the seroprevalence of Helicobacter pylori infection is high in dyspeptic patients attending this primary care setting. Improving living standards and establishing national guidelines for eradication can possibly aid the control of this infection.

## Background

Helicobacter pylori (*H. pylori*) infection is extremely common worldwide with evidence from a recent systematic review suggesting that almost half of the world’s population is infected [1]. Infection rates are higher in resource-poor settings and developing countries, with prevalence rates above 70% reported in Africa, the highest worldwide [1]. The high prevalence in developing countries, has been associated with overcrowding, poor housing, poor sanitation, and unclean water supplies [2–4]. In Cameroon and around the world, infection with *H. pylori* is associated with several upper gastrointestinal diseases including; gastritis, gastric ulcers, duodenal ulcers and gastric cancers [5, 6].

Several methods exist for detecting the infection. Serologic *H. pylori* detection tests are non-invasive and are based on finding antibodies to the bacteria in serum, saliva or urine. A positive serological test therefore indicates exposure, not an ongoing infection. As such serology is considered inferior to other direct non-invasive tests like the urea breath test and the stool antigen test in clinical practice [7, 8]. However, the sensitivity of serological tests for detecting *H. pylori* active infection has been found to range from 50 to 100% and specificity of 30 to 100% [9, 10]. They are considered reliable predictors of the presence of infection in high prevalence settings [11]. Being simple, inexpensive and affordable, they give a good idea of the burden of the infection in the population, especially in low-income settings known to carry the greatest infection rates.

Dyspepsia according to the joint American College of Gastroenterology (ACG) and the Canadian Association of Gastroenterology (CAG) guidelines is; predominant epigastric pain lasting at least 1 month, associated with any other upper gastro intestinal symptom such as epigastric fullness, nausea, vomiting, or heartburn [8]. The United Kingdom, National Institute of Health and Care Excellence defines it more broadly as; any symptom of the upper gastrointestinal tract, present for 4 weeks or more, including upper abdominal pain or discomfort, heartburn, acid reflux, nausea, or vomiting [12]. Dyspeptic individuals are over two times more likely to be *H. pylori* positive, than asymptomatic persons [13]. In addition, dyspepsia is usually the first warning sign for conditions such as peptic ulcers and gastric cancers [14]. The ACG and the World Gastroenterology Organization (WGO) recommend non-invasive *H. pylori* testing for dyspeptic individuals below 60 years of age and the treatment of persons who test positive [8, 11]. First-line and salvage therapy recommended by these two bodies, consists of a combination of a proton pump inhibitor (PPI) with 2 antibiotics; clarithromycin or levofloxacin, associated to amoxicillin or metronidazole. Quadruple therapy of bismuth salicylate and a PPI, associated with two antibiotics; tetracycline plus metronidazole or clarithromycin plus amoxicillin is equally recognized [8, 11].

Focus on understanding the factors associated with *H. pylori* seropositivity in dyspeptic persons and the trends in seroprevalence can provide a guide for public health policies especially in resource poor settings.

In Tanzania, seroprevalence of *H. pylori* was 39.1% in dyspeptic patients [15]. Fluctuating trends in seroprevalence in dyspeptic patients were observed in Ethiopia [16, 17]. In Cameroon, the seroprevalence in symptomatic patients in hospital settings in the North West region was found to be 27.5% [18]. Higher prevalence rates have been reported in hospital-based studies where endoscopy and biopsy methods were used for diagnosis. A study conducted at the teaching hospital in Yaoundé, showed a *H. pylori* infection prevalence of 72.5% among symptomatic patients referred for upper gastrointestinal endoscopy [5]. Additionally, Ankouane and colleagues found prevalence rates of 71.2% among patients with atrophic gastritis, 75% among those with follicular gastritis, and 80% among those with intestinal metaplasia [19]. Among HIV patients presenting with gastro-intestinal symptoms, the prevalence of *H. pylori* infection was 50% [20]. A population-based study carried out in two health districts in Cameroon among asymptomatic children (0–10 years) reported a prevalence of 52.3% for stool *H. pylori* antigen, suggesting that infection in our population is acquired at a very early ages [21].

The majority of studies in Cameroon are from the referral or tertiary hospitals, with very limited literature on the prevalence of *H. pylori* infection in primary care settings. Secondly, none of the existing studies have investigated the trend in seroprevalence of this infection over time. We sought to fill this gap by exploring the trend in *H. pylori* seroprevalence and to determine the predictors of seropositivity among people with dyspepsia attending a primary care hospital in the North West region of Cameroon.

## Methods

### Study design and setting

This was a retrospective analysis of patient records from January 2012 to December 2016 at the Wum District Hospital (WDH) in Cameroon. The WDH is a primary care level hospital with inpatient and outpatient services, located in Wum town, a rural area and third biggest town in the North West region with over 80,000 inhabitants. The town is about 80 km to the north of Bamenda, the regional capital.

### Sampling and study participants

The minimum sample size required for this study was calculated using an online sample size calculator [22]. Considering a population size of 80,000 inhabitants, a margin of error of 0.05, confidence level 95%, and prior prevalence estimate of 27.5% based on a study conducted in the Northwest region [18], a sample of 306 was estimated. We consecutively included all records for adult patients for whom an *H. pylori* serology test was requested during the period of January 2012 to December 2016, the predominant complain being epigastric pain. All patients had at least one upper gastrointestinal symptom reported, including; belching, vomiting, postprandial fullness. Duration of symptoms was not taken into account, as this was absent in the records. We excluded records of patients for whom *H. pylori* serology results were unavailable.

### Study procedures and data collection

The study was approved by the ethical committee of the Wum District Hospital prior to commencement. Data was collected from the outpatient consultation registers of the hospital. Consultation registers or logbooks contain the patient identification, sex, age, symptoms at presentation, presumptive diagnosis, laboratory investigations and results for all outpatients. The logbooks were carefully examined for the periods of January 2012 to December 2016 and all patients for whom *H. pylori* serology was ordered with available results were included in the study.

### Antibody screening

*H. pylori* testing was done using a one-step rapid diagnostic test (DiaSpot®) that utilises immunochromatographic techniques to detect the presence of anti-*H. pylori* (IgG) antibodies in serum or plasma. The appearance of two colour bands (both test and control lines) on the device cassette is considered a positive test result. The test is considered negative if a colour band appears only on the control band, and invalid if a control band fails to appear.

### Study variables

Using a predesigned data collection form, the following data were collected: socio-demographic characteristics including gender, age, marital status, employment status, level of education and religion. Clinical variables included postprandial fullness or early satiety, heartburn, acid regurgitation, excessive belching, nausea, vomiting and HIV status. The dependent variable was a positive *H. pylori* test.

### Data management and statistical analysis

Data were analysed using the Statistical Package for Social Sciences (SPSS Inc., Chicago, Illinois, USA) IBM version 20. Results are summarized as counts and percentages for categorical variables and as means and standard deviation (SD) for continuous variables. The Cochran-Armitage test was used to test for the existence of a significant linear trend in *H. pylori* seroprevalence across the years, and a linear regression model was used to determine the slope (quantify the change) in seroprevalence over time. Basic and multivariable logistic regression models were fitted to investigate the factors associated with *H. pylori* seropositivity. Variables with a *p*-value < 0.1 were included in the multivariable model and adjusted for age and sex. A probability < 0.05 was considered statistically significant.

## Results

### General characteristics of study population

We included 451 records, 63.6% (*n* = 287) of whom were female. The mean age of the study population was 40.7 ± 19.1 (range: 7–96), and 22.8% (*n* = 103) were aged between 15 to 24 years. Employed persons made up 65.2%, while only 21.7% were unemployed or retired. The majority (76.1%) were Christians. Common accompanying symptoms for dyspepsia were belching (16.0%) and vomiting (14.4%) (Table 1).

**Table 1:** Univariate analysis of sociodemographic characteristics associated with *H. pylori* seropositivity

Variable	Frequency (%)	*H. pylori* positive	Odds ratio (95% CI)	*P*-value
Sex (*N* = 451)				
Male	164 (36.4)	85 (51.8)	Ref	
Female	287 (63.6)	147 (51.2)	0.97 (0.67–1.43)	0.901
Age categories, in years (*N* = 451)				
< 15	16 (3.5)	8 (50.0)	Ref	
15–24	103 (22.8)	53 (48.5)	1.06 (0.37–3.04)	0.914
25–34	75 (16.6)	34 (51.5)	0.83 (0.28–2.44)	0.734
35–44	74 (16.4)	43 (45.3)	1.39 (0.47–4.10)	0.554
45–54	69 (15.3)	35 (50.7))	1.03 (0.35–3.06)	0.958
55–64	49 (10.9)	29 (59.2)	1.45 (0.47–4.51)	0.521
**>** 65	65 (14.4)	30 (46.2)	0.86 (0.29–2.56)	0.783
Marital status (*N* = 161)				
Married	103 (64.0)	55 (53.4)	Ref	
Single	32 (19.9)	13 (40.6)	0.60 (0.27–1.34)	0.209
Widow	26 (16.1)	11 (42.3)	0.64 (0.27–1.53)	0.314
Educational level (*N* = 87)				
Secondary + Tertiary	31 (35.6)	17 (54.8)	Ref	
Never + Primary	56 (64.4)	42 (75.0)	2.47 (0.97–6.27)	0.057
Religion (*N* = 451)				
Christian	343 (76.1)	184 (53.6)	Ref	
Muslim	108 (23.9)	48 (44.4)	0.69 (0.45–1.07)	0.096
Employment status (*N* = 161)				
Employed	105 (65.2)	59 (56.2)	Ref	
Unemployed or retired	35 (21.7)	12 (34.3)	0.41 (0.18–0.90)	0.027^a^
Student	21 (13.1)	6 (28.6)	0.31 (0.11–0.87)	0.025^a^
HIV status (*N* = 119)				
Negative	105 (88.2)	47 (44.8)	Ref	
Positive	14 (11.8)	4 (28.6)	0.49 (0.15–1.68)	0.257

^a^Statistically significant, *HIV* = human immunodeficiency virus, *Ref* = reference category

### *H. pylori* seroprevalence and trend over the years

Overall, 51.5% (95% CI: 47–56%) of the study participants were *H. pylori* seropositive. The prevalence of *H. pylori* infection was higher in females than in males (32.6% vs. 18.8%).

**Fig. 1:**
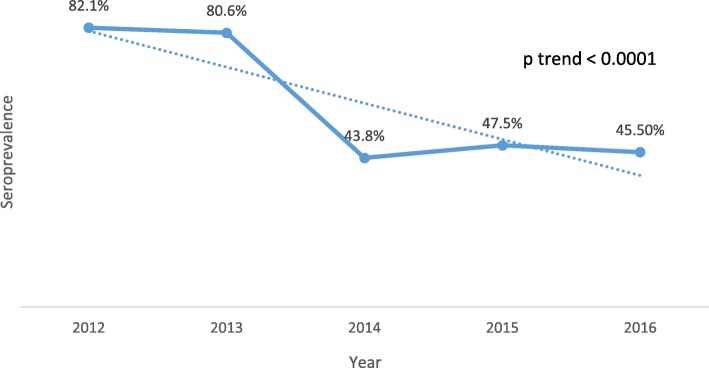
Trends in *H. pylori* seroprevalence at Wum District Hospital, Cameroon

Generally, there was a decline in the seroprevalence of *H. pylori* infection over the five years of the study, from 82.1% in 2012 to 45.5% in 2016. Seroprevalence was decreasing at an average rate of 6.8% annually (*p* trend < 0.0001) (Fig. 1).

### Treatment profile of *H. pylori* positive patients

**Fig. 2:**
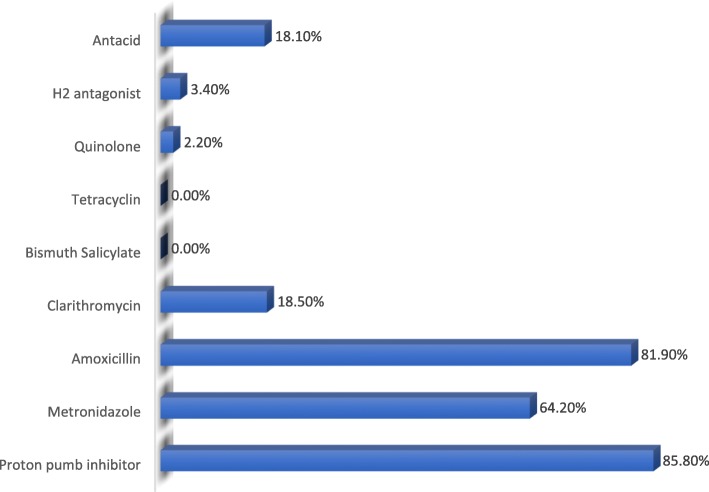
Treatment profile for *H. pylori* positive patients at Wum District Hospital, Cameroon

**Fig. 3:**
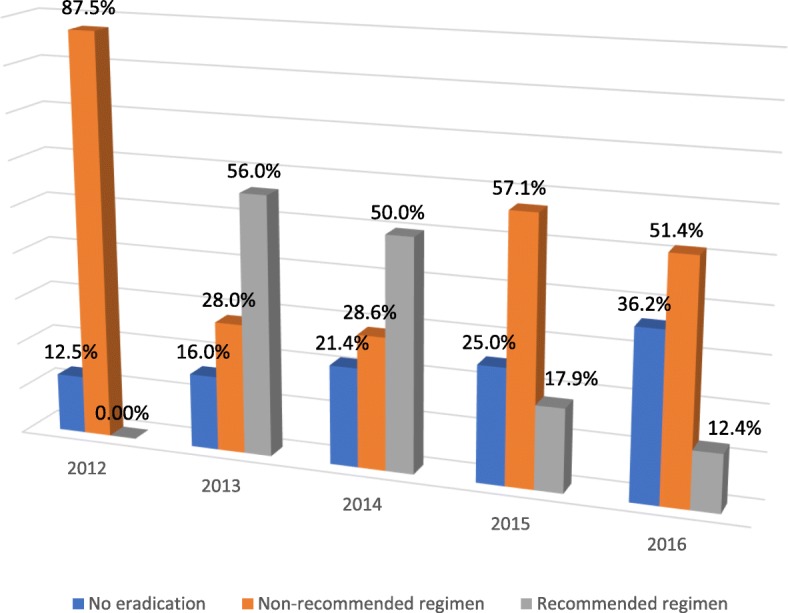
Prescription of *H. pylori* eradication therapy at the Wum District Hospital, Cameroon

Overall, 85.8% of *H. pylori* positive patients received a proton pump inhibitor (PPI) (Fig. 2). As seen in Fig. 3, majority of *H. pylori* positive patients in this study, went without eradication therapy, or received a non-recommended drug combination.

### Factors associated with *H. pylori* seropositivity

In univariate logistic regression analysis; being unemployed and being a student decreased the likelihood of being seropositive (odds ratio [OR] = 0.41, 95% confidence interval [CI]: 0.18–0.90, *p* = 0.027 and OR = 0.31, 95% CI: 0.11–0.87, *p* = 0.025, respectively). Lower education levels tended to increase likelihood of being *H. pylori* seropositive (OR = 2.47, 95% CI: 0.97–6.27, *P* = 0.057). In addition, persons who reported heartburn tended to have a higher likelihood for *H. pylori* infection than their counterparts did (OR = 1.77, 95% CI: 0.94–3.30, *p* = 0.075) (Table 2).

**Table 2:** Univariate analysis of gastrointestinal symptoms associated with *H. pylori* seropositivity

Variable	Frequency (%)	*H. pylori* positive (%)	Odds ratio (95% CI)	*P* value
Nausea (*N* = 451)				
Absent	422 (93.6)	213 (50.5)	Ref	
Present	29 (6.4)	19 (65.5)	1.86 (0.85–4.10)	0.122
Vomiting (*N* = 451)				
Absent	386 (85.6)	199 (51.6)	Ref	
Present	65 (14.4)	33 (50.8)	0.96 (0.57–1.64)	0.907
Belching (*N* = 451)				
Absent	379 (84.00)	189 (49.9)	Ref	
Present	72 (16.0)	43 (59.7)	1.49 (0.89–2.49)	0.127
Heartburn (*N* = 451)				
Absent	404 (89.6)	202 (50.0)	Ref	
Present	47 (10.40)	30 (63.8)	1.77 (0.94–3.30)	0.075
Acid regurgitation (*N* = 451)				
Absent	444 (98.9)	230 (51.6)	Ref	
Present	5 (1.10)	2 (40.0)	0.63 (0.10–3.78)	0.610
Postprandial fullness/early satiety (*N* = 451)				
Absent	447 (99.1)	230 (51.5)	Ref	
Present	4 (0.90)	2 (50.0)	0.94 (0.13–6.76)	0.954

*Ref* = reference category, *CI* = confidence interval

In multivariable regression analysis, only employment status independently predicted seropositivity. Students were less likely to be *H. pylori* positive when compared to employed persons (aOR = 0.09, 95% CI: 0.02–0.49, *p* = 0.005) (Table 3).

**Table 3:** Multivariable analysis showing independent predictors of *H. pylori* seropositivity

*N* = 85	Adjusted OR (95% CI)	*P* value
Age, per year	0.99 (0.96–1.02)	0.373
Sex		
Male	Ref	
Female	1.27 (0.44–3.69)	0.658
Religion		
Christian	Ref	
Muslim	1.40 (0.31–6.39)	0.668
Level of Education		
Secondary + Tertiary	Ref	
Never + Primary	0.86 (0.23–3.20)	0.817
Employment		
Employed	Ref	
Unemployed or retired	0.71 (0.11–4.46)	0.710
Student	0.09 (0.02–0.49)	0.005^a^
Heartburn		
Absent	Ref	
Present	0.74 (0.17–3.21)	0.686

^a^Statistically significant, *OR* = odds ratio, *CI* = confidence interval

## Discussion

This study aimed at describing the trends in seroprevalence of Helicobacter pylori infection, and to identify factors associated with seropositivity in dyspeptic patients attending a primary care hospital in Cameroon. Seroprevalence was high, with over half of study participants infected. We further observed a progressive decline in the seroprevalence rate during the study period (2012–2016), as well as poor and declining use of eradication therapy. Being a student independently predicted a reduced likelihood of testing positive for *H. pylori*.

Our findings reveal a high seroprevalence for *H. pylori* in dyspeptic patients (more than half) attending the Wum District Hospital. The prevalence of *H. pylori* has been known to vary widely across and even within countries. Most studies in symptomatic populations in Africa report prevalence rates that range from 25 to 75%. For example, the seroprevalence in the Tubah District hospital, also in the North West region of Cameroon was comparable (60%) to that in our study [4]. On the other hand, comparatively low rates have also been described, with pooled prevalence of 27.5% reported from four hospitals in Cameroon [18]. This study however was conducted within the last two years of our study period, and their low prevalence rate may reflect a general declining trend in prevalence over the years.

Elsewhere, similar low prevalence rates have been reported. In Uganda, the seroprevalence among dyspeptic patients was 29.9% [23] and histologic prevalence was 36% [24]. Similar lower seroprevalence rates have been observed in Tanzania [15] and Ethiopia [16]. However, other studies in dyspeptic patients in Ethiopia and Sudan have reported higher prevalence rates than in our study population [17, 25–27]. These differences are probably due to geographical and sociodemographic variations between these populations.

While our prevalence rate of 51.5% is high, it is lower than the estimated African prevalence rate of above 70% [1]. It is equally lower than prevalence rates in symptomatic patients who underwent biopsy in Yaoundé, the capital city of Cameroon [5]. The difference probably reflects the higher sensitivity of the biopsy-based rapid urease test (RUT), used as diagnostic tool in the latter study. It could equally be explained by differences in the study settings, as unlike ours, conducted at a primary care setting, the Yaounde study was carried out in a tertiary hospital receiving patients with advanced disease or highly suspicious cases for which prior testing has been done in peripheral hospitals.

During the last decade, while several studies in Africa have described fluctuating trends in *H. pylori* seropositivity [16, 17], this has not been very much the same elsewhere, with a study in Iran reporting a constant trend from 2010 to 2015 [28].

Although prevalence rates were generally high throughout, *H. pylori* seroprevalence appears to be on the decline at our study hospital. Declining trends in infection have equally been reported in some Iranian studies that utilized endoscopic diagnostic methods [29, 30]. Interestingly, the declining seroprevalence trend observed in our study did not reflect prescription of eradication therapy. In our study, there was dwindling use of recommended eradication therapy in *H. pylori* positive patients over the years. This is a major cause for concern as the WGO recommends a test and treat strategy for dyspeptic patients in high prevalence settings, to prevent progression to gastric cancers [11]. This is of particular importance especially for low resource settings like Cameroon with limited resources to cope with the burden associated with cancers. Moreover, the extensive use of non-recommended combination therapy observed in our study, could result in eradication failure and increasing antimicrobial resistance, a current and major public health issue. The declining trends in *H. pylori* seropositivity over the years, despite poor use of eradication therapy could potentially be related to improved standards of living in our study population.

Increasing age is thought to be associated with higher *H. pylori* prevalence rates. This age association has been reported in prior studies in Cameroon [4], Tanzania [15] and Ethiopia [16, 17, 27]. In our study population, even though *H. pylori* seroprevalence was highest in persons 55–64 years old, age did not significantly influence seropositivity. Our results are on par with findings from Uganda and Sudan [23, 25]. With respect to gender, the relationship with *H. pylori* infections is ambiguous in the existing literature. Some studies report significantly higher infection rates in males [16, 31], while some report higher rates in females [4, 31, 32]. In others, as in our study, gender did not significantly influence seropositivity [15, 17, 23, 25, 27].

Low socioeconomic status is a proven risk factor for *H. pylori* infection [21, 27, 33, 34], though some studies have found no association between educational level or employment status with *H. pylori* seropositivity [15, 24–26]. In the present study, employment status significantly influenced seropositivity in univariate analysis, while educational level tended towards significance. The odds of having *H. pylori* infection was higher in dyspeptic individuals with lower educational levels, which is consistent with findings in other settings [15, 23, 35]. Students and the unemployed were less likely to be seropositive when compared with the employed. After controlling for confounders in multipredictor analysis, being a student was the only independent predictor of seropositivity. This may appear to contradict previous studies which suggest that infection with *H. pylori* is associated with lower economic status. However, our study was carried out in a rural community where most persons are employed in subsistent farming, and thus our employment category may not imply better socioeconomic standing. On the other hand, being a student potentially reflects a high educational level, and hence the tendency towards lower infection rates. However, an Asian study equally reported an inverse relationship between employment status and seropositivity [35]. We found no relationship between *H. pylori* seropositivity and HIV status. This was in line with a previous study conducted at a teaching hospital in Cameroon, which found no relationship between HIV seropositivity and *H. pylori* infection rates [20]. None of the associated symptoms including nausea, vomiting, belching, postprandial fullness/early satiety, acid regurgitation and heartburn predicted *H. pylori* seropositivity in our study population. In other settings, gastrointestinal symptoms in dyspeptic patients such as; vomiting, heartburn, postprandial fullness and early satiety had no influence on infection status [23, 24, 35]. However, heartburn and belching have been found associated with seropositivity in Sudan [25] and Iran [36] respectively.

Our study had some limitations that must be discussed. The retrospective nature of our study exposed us to some bias, including potential for misclassification. Moreover, our data source was not particularly designed for this study; hence, we may have lost some information due to incompletely filled records and or missing data. We also could not control for other potential confounders such as previous antibiotic exposure or previous eradication therapy. Secondly, being a hospital-based study, our findings are unlikely to be representative of the general population but probably reflect the pattern at the study hospital. In addition, the cross-sectional design as well as the absence of appropriate control population for comparison limits the possibility to determine causality. Large representative community-based and cohort studies of randomly selected individuals could provide a better depiction of the situation with *H. pylori* infection in our population and its evolution over time. Third, serology techniques as used in this study, may overestimate the true burden of infection, because of high rate of false positives, as opposed to endoscopic and histopathological tests with better diagnostic accuracy. However, in primary care rural settings in low-resourced countries like ours, there is limited availability of such investigations, which are by the way expensive and unaffordable. Hence, serological tests which are cheaper, affordable and less invasive are a good alternative. Despite these shortcomings, our study is among the few to be conducted in primary care settings in Cameroon and the first to demonstrate the trend in seroprevalence of *H. pylori* infection and therapeutic profile of affected individuals. These findings are thus relevant as they inform public health authorities to strengthen efforts towards eradicating this germ and thereby reduce the associated morbidity and mortality.

## Conclusion

Helicobacter pylori infection prevalence is high among dyspeptic patients attending the Wum District Hospital. While trends are decreasing, reflecting positive advancement in this area; improving living standards via education and employment can possible accelerate this process in Cameroon. In addition, adopting the recommendations of the World Gastroenterology Organization, of ‘test and treat’ dyspeptic patients as national guidelines, as well as subsidizing costs and scaling up prescription of standard *H. pylori* eradication therapy would be beneficial in eliminating this germ and stem the associated burden.

## Abbreviations

ACG: American College of Gastroenterology
*H. pylori*: Helicobacter pylori
HIV: Human immunodeficiency virus
OR: Odds ratio
PPI: Proton pump inhibitor
WDH: Wum District Hospital
WGO: World Gastroenterology Organization

## References

[1] Hooi JKY, Lai WY, Ng WK, Suen MMY, Underwood FE, Tanyingoh D (2017). Global prevalence of helicobacter pylori infection: systematic review and meta-analysis. Gastroenterology.

[2] Awuku YA, Simpong DL, Alhassan IK, Tuoyire DA, Afaa T, Adu P (2017). Prevalence of helicobacter pylori infection among children living in a rural setting in sub-Saharan Africa. BMC Public Health.

[3] Bruce MG, Maaroos HI (2008). Epidemiology of helicobacter pylori infection. Helicobacter.

[4] Abongwa LE, Elvis M (2017). Assessing prevalence and risk factors of helicobacter pylori infection in the northwest region of Cameroon. Clin Microbiol.

[5] Andoulo FA, Noah DN, Tagni-Sartre M, Ndam EC Blackett KN. Epidémiologie de l’infection à Helicobacter pylori à Yaoundé: de la particularité à l’énigme Africaine. Pan Afr Med J. 2013;16:115. 10.11604/pamj.2013.16.115.3007 PMC3998896 24778752

[6] Agha A, Graham DY (2005). Evidence-based examination of the African enigma in relation to helicobacter pylori infection. Scand J Gastroenterol.

[7] Ricci C, Holton J, Vaira D (2007). Diagnosis of helicobacter pylori: invasive and non-invasive tests. Best Pract Res Clin Gastroenterol.

[8] Moayyedi PM, Lacy BE, Andrews CN, Enns RA, Howden CW, Vakil N (2017). ACG and CAG clinical guideline: Management of Dyspepsia. Am J Gastroenterol.

[9] Leal YA, Flores LL, Garcia-Cortes LB, Cedillo-Rivera R, Torres J. Antibody-based detection tests for the diagnosis of Helicobacter pylori infection in children: a meta-analysis. Plos One. 2008;3(11):e3751. 10.1371/journal.pone.0003751 PMC2582133 19015732

[10] Khalifehgholi M, Shamsipour F, Ajhdarkosh H, Daryani NE, Pourmand MR, Hosseini M (2013). Comparison of five diagnostic methods for helicobacter pylori. Iran J Microbiol.

[11] Hunt RH, Xiao SD, Megraud F, Leon-Barua R, Bazzoli F, van der Merwe S (2011). Helicobacter pylori in developing countries. World gastroenterology organisation global guideline. J Gastrointest Liver Dis JGLD.

[12] National Institute for Health and Care Excellence (2015). Dyspepsia and gastro-oesophageal reflux disease in adults. NICE.

[13] Suvak B, Dulger AC, Suvak O, Aytemiz E, Kemik O, Suvak B (2015). The prevalence of helicobacter pylori among dyspeptic patients in an earthquake-stricken area. Clinics.

[14] Olokoba AB, Obateru OA, Bojuwoye MO, Ibrahim OK, Olokoba LB (2013). That dyspepsia in the young could be cancer. Niger Med J.

[15] Jaka H, Mushi MF, Mirambo MM, Wilson L, Seni J, Mtebe M (2016). Sero-prevalence and associated factors of helicobacter pylori infection among adult patients with dyspepsia attending the gastroenterology unit in a tertiary hospital in Mwanza, Tanzania. Afr Health Sci.

[16] Workineh M, Andargie D (2016). A 5-year trend of helicobacter pylori seroprevalence among dyspeptic patients at Bahir Dar Felege Hiwot referral hospital, Northwest Ethiopia. Res Rep Trop Med.

[17] Mathewos B, Moges B, Dagnew M (2013). Seroprevalence and trend of helicobacter pylori infection in Gondar University hospital among dyspeptic patients, Gondar, north West Ethiopia. BMC Res Notes.

[18] Abongwa LE, Samje M, Sanda AK, Signang A, Elvis M, Bernadette L (2017). Knowledge, practice and prevalence of helicobacter pylori infection in the north west region of Cameroon. Clin Biotechnol Microbiol.

[19] Ankouane F, Noah DN, Enyime FN, Ndjollé CM, Djapa RN, Nonga BN (2015). Helicobacter pylori and precancerous conditions of the stomach: the frequency of infection in a cross-sectional study of 79 consecutive patients with chronic antral gastritis in Yaoundé, Cameroon. Pan Afr Med J.

[20] Andoulo FA, Kowo M, Ngatcha G, Ndam AN, Awouoyiegnigni B, Sida MB (2016). Prevalence of helicobacter pylori prevalence and upper gastrointestinal endoscopy in HIV/AIDS patients with gastrointestinal symptoms in the university teaching hospitals in Cameroon. Med Sante Trop.

[21] Ndip RN, Malange AE, Akoachere JFT, MacKay WG, Titanji VPK, Weaver LT (2004). Helicobacter pylori antigens in the faeces of asymptomatic children in the Buea and Limbe health districts of Cameroon: a pilot study. Tropical Med Int Health.

[22] Dean AG, Sullivan KM, Soe MM. OpenEpi: Open Source Epidemiologic Statistics for Public Health, Version. www.openepi.com, updated 2013/04/06, Accessed 11/2018/08.

[23] Tsongo L, Nakavuma J, Mugasa C, Kamalha E. Helicobacter pylori among patients with symptoms of gastroduodenal ulcer disease in rural Uganda. Infect Ecol Epidemiol. 2015;5:26785. 10.3402/iee.v5.26785 PMC4641891 26560860

[24] Oling M, Odongo J, Kituuka O, Galukande M (2015). Prevalence of helicobacter pylori in dyspeptic patients at a tertiary hospital in a low resource setting. BMC Res Notes.

[25] Abdallah TM, Mohammed HB, Mohammed MH, Ali AAA (2014). Sero-prevalence and factors associated with helicobacter pylori infection in eastern Sudan. Asian Pac J Trop Dis.

[26] Tadesse E, Daka D, Yemane D, Shimelis T. Seroprevalence of Helicobacter pylori infection and its related risk factors in symptomatic patients in southern Ethiopia. BMC Res Notes. 2014;7:834. 10.1186/1756-0500-7-834 PMC4255656 25421746

[27] Abebaw W, Kibret M, Abera B (2014). Prevalence and risk factors of H. pylori from dyspeptic patients in Northwest Ethiopia: a hospital based cross-sectional study. Asian Pac J Cancer Prev.

[28] Salehi M, Ghasemian A, Mostafavi S, Khalil S, Najafi S, Rajabi Vardanjani H (2017). Sero-prevalence of helicobacter pylori infection in Neyshabur, Iran, during 2010-2015. Iran J Pathol.

[29] Farshad S, Japoni A, Abdolvahab A, Zarenezhad M, Ranjbar R (2010). Changing prevalence of helicobacter pylori in south of Iran. Iran J Clin infect dis J. Clin Infect Dis.

[30] Ashtari S, Pourhoseingholi MA, Molaei M, Taslimi H, Zali MR (2015). The prevalence of helicobacter pylori is decreasing in Iranian patients. Gastroenterol Hepatol Bed Bench.

[31] Alazmi WM, Siddique I, Alateeqi N, Al-Nakib B (2010). Prevalence of helicobacter pylori infection among new outpatients with dyspepsia in Kuwait. BMC Gastroenterol.

[32] Zevit N, Niv Y, Shirin H, Shamir R (2011). Age and gender differences in urea breath test results. Eur J Clin Investig.

[33] Chen H-L, Chen M-J, Shih S-C, Wang H-Y, Lin I-T, Bair M-J (2014). Socioeconomic status, personal habits, and prevalence of helicobacter pylori infection in the inhabitants of Lanyu. J Formos Med Assoc.

[34] Genta RM, Turner KO, Sonnenberg A (2017). Demographic and socioeconomic influences on helicobacter pylori gastritis and its pre-neoplastic lesions amongst US residents. Aliment Pharmacol Ther.

[35] Hamrah MH, Hamrah MS, Hassan Hamrah M, Kanda M, Hamrah AE, Dahi AE (2017). Prevalence of helicobacter pylori infection in dyspeptic patients in Andkhoy Afghanistan. Asian Pac J Cancer Prev APJCP.

[36] Shokrzadeh L, Baghaei K, Yamaoka Y, Shiota S, Mirsattari D, Porhoseingholi A (2012). Prevalence of helicobacter pylori infection in dyspeptic patients in Iran. Gastroenterol Insights.

